# Physico-chemical requirements and kinetics of membrane fusion of flavivirus-like particles

**DOI:** 10.1099/vir.0.000113

**Published:** 2015-07

**Authors:** Danillo L. A. Espósito, Jennifer B. Nguyen, David C. DeWitt, Elizabeth Rhoades, Yorgo Modis

**Affiliations:** ^1^​ Department of Molecular Biophysics & Biochemistry, Yale University, 266 Whitney Avenue, New Haven, CT 06520, USA; ^2^​ Department of Physics, Yale University, New Haven, CT 06520, USA

## Abstract

Flaviviruses deliver their RNA genome into the host-cell cytoplasm by fusing their lipid envelope with a cellular membrane. Expression of the flavivirus pre-membrane and envelope glycoprotein genes in the absence of other viral genes results in the spontaneous assembly and secretion of virus-like particles (VLPs) with membrane fusion activity. Here, we examined the physico-chemical requirements for membrane fusion of VLPs from West Nile and Japanese encephalitis viruses. In a bulk fusion assay, optimal hemifusion (or lipid mixing) efficiencies were observed at 37 °C. Fusion efficiency increased with decreasing pH; half-maximal hemifusion was attained at pH 5.6. The anionic lipids bis(monoacylglycero)phosphate and phosphatidylinositol-3-phosphate, when present in the target membrane, significantly enhanced fusion efficiency, consistent with the emerging model that flaviviruses fuse with intermediate-to-late endosomal compartments, where these lipids are most abundant. In a single-particle fusion assay, VLPs catalysed membrane hemifusion, tracked as lipid mixing with the cellular membrane, on a timescale of 7–20 s after acidification. Lipid mixing kinetics suggest that hemifusion is a kinetically complex, multistep process.

## Introduction

Flaviviruses, such as dengue (DENV), West Nile (WNV), Japanese encephalitis (JEV) and tick-borne encephalitis (TBEV) viruses, are important human pathogens (Lindenbach *et al.*, 2013). Flaviviruses are transmitted between their vertebrate hosts by either mosquitoes or ticks (Lindenbach *et al.*, 2013). Flavivirus entry into host cells is contingent upon multiple processes, including receptor binding, membrane fusion, genome release and virus replication. Although flaviviruses are generally thought to enter the cell via clathrin-mediated endocytosis, several alternative entry pathways have been proposed for specific viruses and cell types (Acosta *et al.*, 2009; Li *et al.*, 2012). Fusion of the viral and endosomal membranes, a key step in virus entry, is catalysed by the envelope glycoprotein, E. In the current model of membrane fusion, E responds to the reduced endosomal pH with a series of conformational rearrangements, which expose a hydrophobic fusion motif, insert the fusion motif into the endosomal membrane, bend the viral and cellular membranes towards each other, induce a hemifusion intermediate in which the outer apposed lipid leaflets of the two membranes are fused and, finally, generate a fusion pore to complete the fusion process (Kielian, 2006; Modis, 2014).

Various compartment-specific lipids promote membrane fusion of DENV and JEV (Das *et al.*, 2010; Nour *et al.*, 2013; Tani *et al.*, 2010; Zaitseva *et al.*, 2010; Zhu *et al.*, 2012). Anionic lipids, such as phosphatidylserine (PS), are particularly important for efficient fusion of flaviviruses (Zaitseva *et al.*, 2010). PS is abundant in early endosomal compartments. The anionic lipid bis(monoacylglycero)phosphate (BMP) is required for yellow fever virus infectivity, ensuring correct intracellular trafficking of the virus (Nour *et al.*, 2013). BMP is present in internal membranes, but not the limiting membrane, of late endosomes (Kobayashi *et al.*, 1998, 1999, 2002; Möbius *et al.*, 2003). BMP regulates endosomal membrane sorting and dynamics by driving the budding of endosomal carrier vesicles (ECVs) from intermediate endosomal membranes, and the back fusion of ECVs to the late endosomal membrane (Le Blanc *et al.*, 2005). Cholesterol in the target membrane also promotes fusion (Gollins & Porterfield, 1986; Stiasny *et al.*, 2003), and cholesterol chelation reduces flavivirus infectivity (Medigeshi *et al.*, 2008). Cholesterol is not strictly required for fusion, however, and addition of exogenous cholesterol at the cellular attachment step can block JEV and DENV cell entry (Lee *et al.*, 2008). Cholesterol is abundant in early endosomal compartments (Möbius *et al.*, 2003), but it is gradually replaced by ceramide as endosomes mature into late endosomes and lysosomes (Schulze *et al.*, 2009).

Imaging of fluorescently labelled flaviviruses during cell entry, in combination with the lipid requirements of flavivirus fusion described above, indicate a cell-entry mechanism in which the virus particles fuse preferentially with ECVs, delivering the particle contents into the ECV lumen (Nour *et al.*, 2013; van der Schaar *et al.*, 2008). In order for the viral nucleocapsid to be delivered into the cytoplasm, the ECVs must then fuse back to the limiting membrane of the late endosome. This back fusion depends on the lipid BMP (Le Blanc *et al.*, 2005; Nour *et al.*, 2013). This cell-entry pathway is very similar to that proposed previously for vesicular stomatitis virus (Le Blanc *et al.*, 2005). Flavivirus virus-like particles (VLPs), which are released during natural infection and have shown promise as vaccine candidates (Davis *et al.*, 2001; Konishi & Fujii, 2002; Konishi *et al.*, 1992, 1998), follow the same pathway (Nour *et al.*, 2013). A recent study established the kinetic and mechanistic framework of flavivirus hemifusion (Chao *et al.*, 2014). The lipid and other physico-chemical requirements for virus and VLP fusion have not been described in detail, however. In this study, we used a combination of bulk and single-particle membrane fusion assays to identify the key physico-chemical requirements for fusion of WNV and JEV VLPs to liposomal target membranes, and to obtain some basic kinetic parameters of the fusion process.

## Results and Discussion

### Virus particles are more fusogenic at higher temperatures

**Fig. 1.**
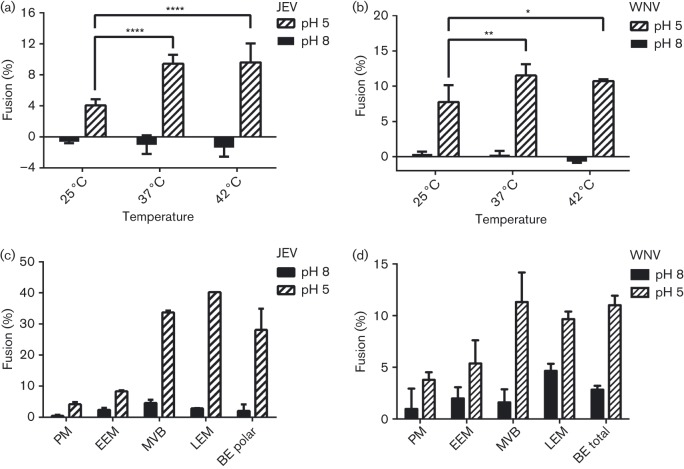
Membrane fusion efficiency of JEV and WNV VLPs as a function of temperature and lipid composition. (a, b) Fusion efficiency, measured as lipid mixing, of JEV (a) and WNV (b) VLPs is greater at 37 and 42 °C than at 25 °C. (c, d) Fusion efficiency of JEV (c) and WNV (d) VLPs with liposomes with compositions mimicking different cellular compartments. PM, plasma membrane; EEM, early endosomal membranes; MVB, multivesicular bodies; LEM, late endosomal membranes; BE, porcine brain extract lipids. Each value is the mean±standard deviation of triplicate measurements.

Hemifusion of WNV and JEV VLPs with lipid vesicles was measured as fluorescence dequenching of the long-chain dialkylcarbocyanine membrane-soluble dye 1,1′-dioctadecyl-3,3,3′,3′-tetramethylindocarbocyanine perchlorate (DiI) upon lipid mixing. Fluorescence after detergent solubilization was used to define 100 % fusion. The VLPs had a mean apparent hemifusion efficiency of 15 % with total brain extract lipids but with considerable variation (5–25 %) among VLP batches. Half-maximal hemifusion was reached approximately 5 min after acidification of the medium, and DiI fluorescence kept increasing for approximately 20 min after acidification. Hemifusion efficiency at 37 °C was nearly twofold higher than at 25 °C (the body temperature of many insects) for both WNV and JEV VLPs. Hemifusion efficiency at 42 °C (the body temperature of many birds) was not significantly different from that at 37 °C. There was no statistically significant fusion activity at pH 8.0 at any of the tested temperatures (Fig. 1a, b).

Mature DENV virions undergo significant structural changes, from a smooth form to a bumpy form, as the temperature is increased from 25 to 37 °C (Fibriansah *et al.*, 2013; Zhang *et al.*, 2013). Electron microscopy (EM) reconstructions show that, in the bumpy form, the E proteins project outward from the viral membrane. This arrangement of E proteins may increase accessibility of E to cellular membranes and receptors, providing a possible explanation for the higher observed infectivity of virions at 37 than at 25 °C (Zhang *et al.*, 2013). It is unclear whether similar structural changes occur in VLPs, because the icosahedral assembly is different in VLPs compared with full-sized virions. WNV VLPs are smaller than mature virions (30 vs 50 nm diameter) (Schalich *et al.*, 1996). An EM structure of TBEV VLPs revealed a *T* = 1 quasi-equivalent icosahedral assembly of 60 copies of E, whereas mature virions have 180 E molecules in a tightly packed, non-equivalent, herringbone arrangement (Ferlenghi *et al.*, 2001). The viral membrane is more exposed in VLPs than in mature virions (Ferlenghi *et al.*, 2001). However, the EM structure is thought to represent only a subset of a heterogeneous VLP population, which may include a VLP subpopulation with an arrangement of E that is more similar to mature virions (Kaufmann *et al.*, 2010). Therefore, it remains unclear whether increased ‘breathing’ or conformational rearrangements in VLPs are responsible for the increased fusion activity at 37 and 42 °C relative to 25 °C. An alternative explanation to account for the fusion enhancement observed at higher temperatures is the increased fluidity of the VLP and liposome membranes at higher temperatures.

### WNV and JEV VLPs require anionic phospholipids for membrane fusion

In addition to temperature, the lipid composition of the membranes affects membrane fluidity and hence fusion activity. To establish the lipid requirements for membrane fusion of JEV and WNV VLPs, we measured low-pH-induced lipid mixing between JEV or WNV VLPs labelled with a self-quenching concentration of DiI and unlabelled liposomes with different lipid compositions (Table 1). Notably, VLPs did not display statistically significant fusion with liposomes with lipid compositions mimicking the outer leaflet of mammalian plasma membranes (PM) or early endosomal membranes (EEMs) (Fig. 1c, d) (Gruenberg, 2003; van Meer *et al.*, 2008). In contrast, efficient hemifusion was observed for VLPs with liposomes with the lipid composition of multivesicular bodies (MVBs). The MVB composition takes into account the composition of ECVs, which contain phosphatidylinositol-3-phophate (PI_3_P) (Gillooly *et al.*, 2000; Gruenberg, 2003; Gruenberg & Stenmark, 2004; Möbius *et al.*, 2003). Moreover, a lipid composition corresponding to late endosomal membranes (LEMs), containing PI_3_P and BMP, which is specific to late endosomes, resulted in similarly efficient hemifusion (Fig. 1c, d) (Kobayashi *et al.*, 1998, 1999, 2002; Möbius *et al.*, 2003; van Meer *et al.*, 2008). As BMP and PI_3_P become enriched in ECVs as endosomes mature (van Meer *et al.*, 2008), these anionic lipids may contribute to productive membrane fusion and cell entry of JEV and other flaviviruses.

**Table 1.** Lipid compositions (%) of synthetic liposome membranes Lipid compositions for different cellular compartments are approximate, and based on the following references: plasma membrane and early endosomal membrane: Gruenberg (2003); van Meer *et al.* (2008); multivesicular bodies: Gillooly *et al.* (2000); Gruenberg (2003); Gruenberg & Stenmark (2004); Möbius *et al.* (2003); late endosomal membranes: Kobayashi *et al.* (2002); Kobayashi *et al.* (1999); Kobayashi *et al.* (1998); Möbius *et al.* (2003); van Meer *et al.* (2008).

Cellular compartment/lipid ratio	Lipid (%)								
	POPC	POPE	POPG	POPS	PI_3_P	BMP	C1P	Chol	Total
Plasma membrane	45	10	–	5	0	–	–	40	100
Early endosomal membrane	45	10	–	15	0	–	–	30	100
Multivesicular bodies	30	20	–	10	10	–	–	30	100
Late endosomal membranes	40	20	–	–	5	15	–	20	100
PC :PG : Chol	40	–	40	–	–	–	–	20	100
PC : PS : Chol	40	–	–	40	–	–	–	20	100
PC : BMP : Chol	40	–	–	–	–	40	–	20	100
PC : C1P : Chol	40	–	–	–	–	–	40	20	100

POPC, 1-palmitoyl-2-oleoyl-*sn*-glycero-3-phosphocholine; POPE, 1-palmitoyl-2-oleoyl-*sn*-glycero-3-phosphoethanolamine; POPS, 1-palmitoyl-2-oleoyl-*sn*-glycero-3-phospho-l-serine; POPG, 1-palmitoyl-2-oleoyl-*sn*-glycero-3-[phospho-rac-(1-glycerol)]; C1P, *N*-palmitoyl-ceramide-1-phosphate; PI_3_P, phosphatidylinositol-3-phophate; BMP, oleoyl bis(monoacylglycero)phosphate (*S,S*) isomer; Chol, cholesterol; PC, phosphatidylcholine; PS, phosphatidylserine; PG, phosphatidylglycerol.

**Fig. 2.**
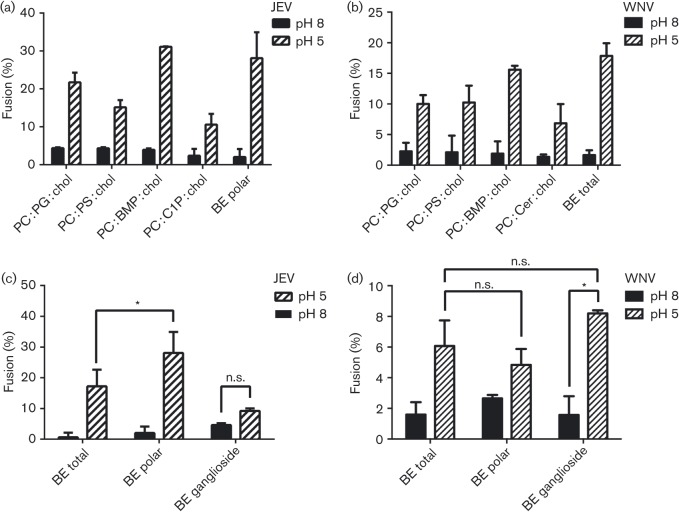
Role of polar and anionic lipids in VLP fusion. (a, b) Fusion efficiency of JEV (a) and WNV (b) VLPs with liposomes containing a ternary mixture of phosphatidylcholine (PC), with BMP, phosphatidylglycerol (PG), phosphatidylserine (PS), ceramide-1-phosphate (C1P) or ceramide (Cer), together and cholesterol (chol) at a 4 : 4 : 2 molar ratio. (c, d) Fusion efficiency of JEV (c) and WNV (d) VLPs with liposomes composed of total porcine brain extract lipids (BE total), polar brain lipids (BE polar) or ganglioside brain lipids (BE ganglioside).

Anionic lipids including BMP, when present at ≥30 % molar fraction in the target membrane, promote membrane fusion of flaviviruses (Zaitseva *et al.*, 2010). To evaluate in more detail the importance of BMP and other anionic lipids in VLP fusion, we measured VLP hemifusion with liposomes containing a mixture of phosphatidylcholine (PC), an anionic lipid and cholesterol at a 4 : 4 : 2 molar ratio. BMP, phosphatidylglycerol, PS, ceramide and ceramide-1-phosphate (C1P) were each selected as the anionic lipid in separate experiments. The PC and cholesterol molar ratios were chosen to ensure membrane fluidity and to approximately mimic the membrane composition of an intermediate endosome. The extent of hemifusion observed for the BMP-containing liposomes with VLPs was significantly higher than for liposomes containing other negatively charged lipids in place of BMP (Fig. 2a, b). Although these exact mixtures are not physiologically relevant, these results highlight the activity of BMP in promoting flavivirus membrane fusion.

Ceramides and ceramide phosphates have been implicated in membrane fusion and promotion of membrane invagination in autophagosome formation, owing to their unique, cone-shaped molecular structures, which promote negative spontaneous curvature of lipid vesicles (Goñi & Alonso, 2006; Holopainen *et al.*, 2000). C1P contributes to the regulation of vesicle transport (Bajjalieh *et al.*, 1989). However, ceramide and C1P did not contribute to lipid mixing of WNV and JEV VLPs, respectively. Moreover, the efficient fusion of VLPs to liposomes containing PI_3_P (with MVB and LEM lipid compositions) demonstrated that flaviviruses are capable of distinguishing PI_3_P from ceramides (Fig. 1c, d).

Fusion of synaptic vesicles in response to an action potential is very fast (<0.2 ms) (Sabatini & Regehr, 1996; Zorman *et al.*, 2014). As the lipid composition of synaptic vesicles supports rapid fusion kinetics, we measured hemifusion of VLPs with liposomes formed from total brain extract lipids, which derive to a large extent from synaptic vesicles. VLP hemifusion was similarly efficient to hemifusion with BMP-containing liposomes (Fig. 2c, d). The hemifusion efficiency of JEV VLPs to polar brain lipids, which according to the manufacturer (Avanti Polar Lipids), are enriched in anionic lipids such as PS and phosphatidylinositols (PIs), was slightly higher than for total brain extract, suggesting that the presumably low abundance of BMP in these lipid preparations may be compensated by increases in other anionic lipids. However, the same trend was not observed for WNV VLPs. Interestingly, JEV VLPs were not fusogenic towards gangliosides extracted from brain tissue, whereas WNV VLPs were fusogenic but with a similar extent of fusion to total and polar brain extracts (Fig. 2c, d). Hence, gangliosides, which are anionic lipids, are not responsible for the increased fusion efficiency of JEV VLPs to polar brain lipids. Taken together, these results suggested that flavivirus particles have a clear propensity to fuse with membranes containing lipids found in the intermediate-to-late endocytic pathway, including PI_3_P and BMP.

### JEV and WNV VLPs have a broad pH threshold for fusion

**Fig. 3.**
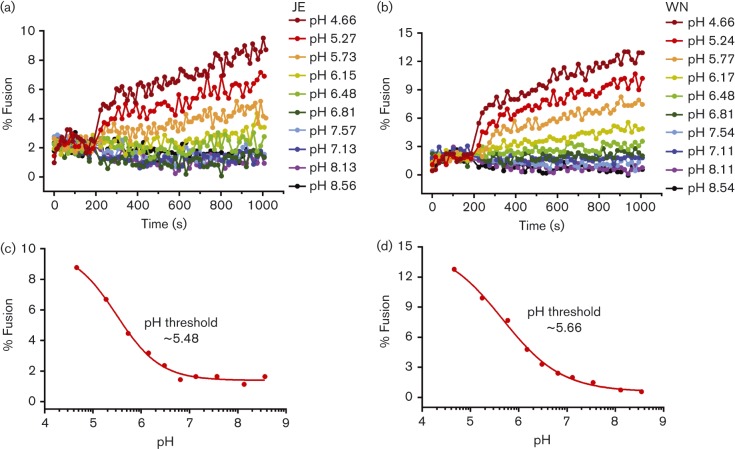
Membrane fusion efficiency of JEV and WNV VLPs as a function of pH. (a, b) Fusion extent as a function of time post-acidification for JEV (a) VLPs and WNV (b) VLPs in SPG buffer (see Methods) at the indicated pH values. Curves were normalized to the fluorescence obtained after detergent solubilization of the self-quenched DiI in the VLPs. (c, d) End-point representations of the curves in (a) and (b), respectively, plotting fusion extent as a function of pH. Half-maximal fusion was observed at ~pH 5.5 for JEV VLPs and ~pH 5.7 for WNV VLPs.

We showed that JEV and WNV VLPs undergo efficient hemifusion with vesicles with lipid compositions similar to the membranes of intermediate and late endosomal compartments but not early endosomal compartments. Late endosomes have a lower luminal pH (pH 5–5.7) than early endosomes (pH 6.3–7). To assess the correlation between pH and fusion potential, we measured VLP hemifusion to brain extract liposomes as a function of pH. Unexpectedly, JEV and WNV VLPs did not have a sharp pH threshold for fusion and displayed increasing hemifusion activity with decreasing pH up to pH 4.5 (Fig. 3). Half-maximal DiI fluorescence was observed at ~pH 5.5 for JEV VLPs and ~pH 5.7 for WNV VLPs, corresponding to the pH of intermediate endosomes. The pH threshold determined previously for WNV was pH 6.0–6.5 (Krishnan *et al.*, 2007; Moesker *et al.*, 2010). The lower pH threshold and broader pH response curve of VLPs versus virions may reflect differences in the assembly and fusion mechanisms in virions and VLPs. Nonetheless, our findings that half-maximal hemifusion occurred at pH 5.5–5.7 with membranes containing BMP and PI_3_P are consistent with recent evidence that certain flaviviruses and flavivirus VLPs fuse predominantly with small ECVs in intermediate endosomes and require a second and distinct fusion event, between the ECV membrane and the limiting late endosomal membrane, for nucleocapsid delivery into the cytoplasm (Fig. S1, available in the online Supplementary Material) (Nour *et al.*, 2013). Back fusion of ECVs is dependent on BMP in the ECV membrane (Le Blanc *et al.*, 2005; Nour *et al.*, 2013).

### Single-particle total internal reflection fluorescence microscopy (TIRFM) assay

**Fig. 4.**
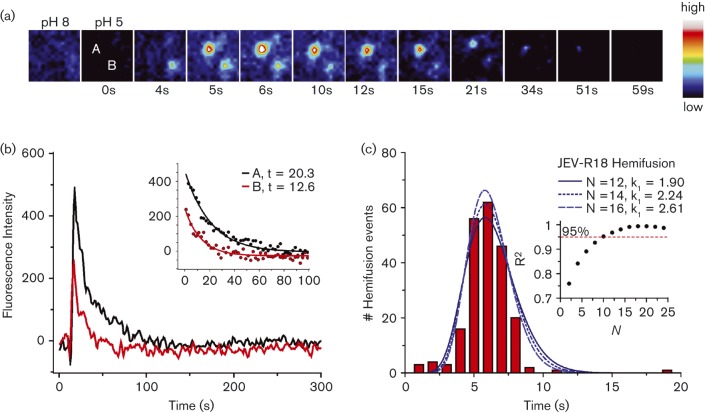
TIRFM of membrane hemifusion by single JEV VLPs. (a) Snapshots (after bilinear interpolation) of two R18-labelled JEV VLPs fusing with the planar membrane. Images were false coloured with the indicated pixel value map. (b) Fluorescence intensities of the particles in (a) over the time course of fusion. Lipid mixing caused sudden dequenching of R18, followed by decay due to diffusion of the dye into the membrane. The exponential decay constant was 13–20 s (inset). (c) Distribution of time elapsed between pH drop and hemifusion of individual particles (*n* = 214). The best fit to a gamma distribution function was with *N* = 15 transitions, *k*_1_ = 2.43 s^−1^. Inset, *R^2^* values of gamma distribution fits at various *N* values.

To measure the fusion kinetics of single virus particles, JEV VLPs were labelled with self-quenching concentrations of the membrane-soluble dye octadecyl rhodamine B chloride (R18). The VLPs were then injected into a microfluidic flow cell containing a fluid target planar membrane. Addition of 0.05 % GD3 ganglioside to the target membrane resulted in loose attachment of VLPs to the membrane. This loose attachment greatly facilitated VLP tracking by TIRFM as attached particles could be identified on the target membrane well in advance of the hemifusion event. Exactly how VLPs bind GD3 ganglioside is unknown, but non-specific binding may occur via interaction of charge-complementary moieties, as the GD3 ganglioside head group is negatively charged and flaviviruses have positively charged patches on their outer surface (Modis *et al.*, 2005). Notably, the positively charged patches on DENV have been proposed to bind to negatively charged heparan sulfate on the cell surface (Chen *et al.*, 1997; Hung *et al.*, 2004). Upon acidification of the medium, mixing of the viral lipids with lipids from the target membrane diluted the R18 dye, resulting in dequenching of R18 fluorescence. Thus, fusing JEV VLPs appeared in the microscope as diffraction-limited R18 fluorescence puncta. Hemifusion occurred on average 7 s after acidification (Fig. 4). This timescale for hemifusion is similar to the kinetics of hemifusion of influenza virus (Floyd *et al.*, 2008). WNV VLPs fused approximately 20 s after acidification (Fig. S2), whereas yellow fever virus (NS1-deficient strain) exhibited a seemingly more stochastic fusion distribution, with most particles fusing within a broad time frame (10–60 s) and some particles fusing up to 130 s after acidification (data not shown). Consistent with these results, a recent independent study reported the hemifusion time for Kunjin virus to be in the range of 29–74 s (Chao *et al.*, 2014). The same study reported the hemifusion time of WNV VLPs at 41–56 s, approximately 2.4-fold slower than in our assays. This difference may be attributable to differences in the lipid composition and pH in the two studies. After hemifusion, R18 fluorescence decayed exponentially as the dye diffused into the membrane, with a first-rate exponential decay constant of 13–20 s (Fig. 4b).

A simple kinetic model was developed to describe the membrane fusion reaction mechanism of influenza virus as a series of *N* transition steps between initial and final states during fusion (Floyd *et al.*, 2008). Each transition was considered as a random (Poisson) process with a single rate constant, *k*_1_. Based on this model, the probability of turnover at a given time *t* was represented as *k*_1_×exp(−*k*_1_ *t*) and the overall hemifusion probability density consisted of the convolution of each intermediate step, which could be represented by a gamma distribution function:

$$P_{H}\left(t\right)=\frac{k_{1}^{N}t^{N-1}}{\Gamma \left(N\right)}e^{-k_{1}t}$$

where *k*_1_ represents the rate constant at a given time *t* for *N* transition steps.

Application of the gamma distribution function to fit the hemifusion data from our single-particle fusion assays with JEV VLP produced values of *k*_1_ = 1.9–2.6 s^−1^ with *N* = 12–16 transitions, with the best fit at N = 15. (Fig. 4c). This suggests that JEV VLP hemifusion proceeds via a mechanism that involves up to 15 distinct kinetic transitions. Given the complexity of the conformational rearrangements required for membrane fusion, it is possible that *N* = 12–16 relates to the number of molecular transitions in E during membrane fusion catalysis, such as fusion loop exposure, dissociation of at least three dimers (Chao *et al.*, 2014), membrane insertion of the fusion loop, formation of at least two trimers (Chao *et al.*, 2014), and fold-back of each E molecule within the two or more trimers required for fusion. We note that Chao *et al.* (2014) reported *N* = 1–2 transitions for hemifusion of Kunjin virus and WNV VLPs using a similar approach. One possible contributing factor for the discrepancy with the *N* = 12–16 value reported here is that our analysis employed a much finer time window in the histogram of hemifusion dwell times [1 s window in Fig. 4(c) versus 17–37 s windows in Chao *et al.* (2014)]. Indeed, the lag period preceding R18 dequenching, which would only be apparent using a 1–3 s time window, is a clear indication that hemifusion in JEV VLPs consists of multiple kinetic steps.

### Conclusions

Flaviviruses include important human and animal pathogens such as DENV, WNV, JEV and TBEV. Recombinant flavivirus-like particles have shown promise as vaccine candidates, and as delivery vehicles for genes or other therapeutic agents. To deliver the viral genome or other cargo into the cell, the viral and cellular membranes must be fused. This study identified specific physico-chemical requirements for membrane fusion of flavivirus particles. Flavivirus VLPs fused most efficiently at 37–42 °C, and fusion activity increased with decreasing pH, with half-maximal activity at pH 5.5–5.7. VLPs had a clear propensity to fuse with membranes containing PI_3_P and BMP, consistent with the model that flaviviruses fuse with intermediate-to-late endosomal compartments, where these lipids are most abundant. We also began to dissect the complex kinetics of the membrane fusion reaction using a single-particle fusion assay. VLP hemifusion occurred within approximately 10 s of a pH reduction below the fusion threshold and involved a complex multistep series of kinetic transitions. It should be noted that the bulk fusion and TIRFM *in vitro* assays used in this study were quite artificial. Both assays employed lipid mixtures that may not accurately reflect the lipid mixture encountered by a flavivirus particle during membrane fusion in the context of a live host cell. Moreover, the target membrane for fusion *in vivo* is likely to have a different curvature than the target membranes used in this study. It is also possible that other proteins or co-factors from the host cell promote fusion. Future studies are therefore necessary to validate and extend the kinetic and physico-chemical parameters of membrane fusion extracted in this study with more physiologically relevant assays.

## Methods

### Expression and purification of recombinant JEV and WNV VLPs.

WNV and JEV VLPs were produced in mammalian cells with the portion of the genome encoding the pre-membrane (prM)–E genes, along with the C-terminal transmembrane helix of the capsid (C) gene, which was included as the secretion signal, were cloned into a mammalian expression plasmid. VLPs were released into the medium for several days after transfection.

WNV VLPs were expressed from a stable CHO cell line kindly donated by Ted Pierson (NIH, Bethesda, MD, USA) containing a plasmid with a tetracycline-inducible promoter and the coding sequence for the WNV structural proteins prM and E. The cells were propagated at 37 °C in F12 medium (Invitrogen) supplemented with 10 % FBS. VLP expression was induced at 90 % confluency in fresh medium with tetracycline (2 µg ml^−1^). After induction, the cell-culture supernatant was harvested in two batches, after 2 days and after 3–5 days, and was stored for up to 2 weeks at 4 °C. The two batches of culture supernatant were combined and centrifuged at 500 ***g*** and 4 °C to remove cells and debris. VLP expression was monitored by Western blotting using a rabbit anti-WNV E antibody (L^2^ Diagnostics LLC) (Gould *et al.*, 2005).

JEV VLPs were expressed in HEK293T cells transiently transfected with a pcDNA3.1(+) plasmid encoding the prM and E genes from JEV strain CH2195LA (GenBank accession no. AF221499), a gift from Suh-Chin Wu (National Tsing Hua University, Taiwan). HEK293T cells were grown in DMEM (Dulbecco's Modified Eagle's Medium) supplemented with 10 % FBS. At 60–80 % confluency, the medium was replaced with Opti-MEM (Invitrogen) and the cells were transfected. At 7 h post-transfection, DMEM plus 10 % FBS was added to the cells. The supernatant was harvested for JEV VLPs in the same manner as for WNV VLPs.

To purify the VLPs, PEG6000 (Sigma-Aldrich) was added to the clarified supernatant to a final concentration of 10 % (w/v) and supplemented with 2.5 % NaCl (w/v). VLPs were pelleted by centrifugation at 40 000 r.p.m. for 2 h in a Ti-45 rotor (Beckman Coulter). The PEG-containing supernatant was discarded, and the pellet was resuspended in 2 ml TE buffer [50 mM Tris/HCl (pH 8.4), 0.1 mM EDTA, 0.15 M NaCl] and centrifuged at 15 000 r.p.m. (Ti-45 rotor) for 2 min to remove insoluble material in the pellet. The solution containing the resuspended VLPs was subsequently loaded onto a 10–60 % continuous sucrose gradient [10 mM Tris/HCl (pH 8.5), 100 mM NaCl] and subjected to ultracentrifugation at 30 000 r.p.m. for 4 h in a SW-32 rotor (Beckman Coulter) at 4 °C. Fractions were collected by bottom puncture and analysed by Western blotting.

VLPs were further purified on a Heparin HP affinity column (GE Biosciences) to remove additional contaminants. Sucrose gradient fractions containing VLPs were loaded onto the column in phosphate buffer [10 mM NaH_2_/Na_2_HPO_4_ (pH 8.3)], washed with 10 column vols phosphate buffer and eluted with a gradient of 0–1 M NaCl. Two peaks were eluted from the column at approximately 0.5 and 1 M NaCl, with the first peak corresponding to the VLPs based on Western blot analysis. The concentration of E protein in the purified VLP solution was measured from the band intensity on a Coomassie blue-stained SDS-PAGE gel. The VLP concentration was calculated assuming 60 E proteins per VLP (Ferlenghi *et al.*, 2001).

### Liposome preparation.

Lipids were purchased from Avanti Polar Lipids and Sigma-Aldrich. Liposomes were prepared from the set of lipid mixtures listed in Table 1. The phospholipid compositions of brain total extract and brain polar extract are listed by Avanti Polar Lipids as follows: total extract, 9.6 % PC, 16.7 % phosphatidylethanolamine (PE), 1.6 % PI, 10.6 % PS, 2.8 % phosphatidic acid (PA) and 58.7 % unknown; polar extract, 12.6 % PC, 33.1 % PE, 4.1 % PI, 18.5 % PS, 0.8 % PA and 30.9 % unknown. Lipid mixtures dissolved in chloroform were dried with argon gas and under vacuum. The resulting lipid film was resuspended in TEA buffer [10 mM triethanolamine (pH 8.3), 0. 14 M NaCl] to a final concentration of 2.5 g lipids l^−1^ and subjected to 10 freeze–thaw cycles using liquid nitrogen and a 37 °C water bath. Unilamellar vesicles were obtained by extrusion through a 100 nm membrane using a lipid extruder (Avanti Polar Lipids).

### Fluorescent labelling of VLPs.

VLPs were labelled with acylated membrane-soluble dyes from Life Technologies. For the single-particle kinetics experiments, VLPs were labelled with R18. For bulk fusion assays, VLPs were labelled with DiI. R18 and DiI were dissolved in 100 % ethanol at a dye concentration of 10 mM. Each dye stock solution was added to a 1 ml VLP suspension containing approximately 2×10^12^ VLPs to a final dye concentration of 30 µM. The dye/VLP mixtures were incubated for 1 h without agitation at 4 °C. VLPs were purified from non-incorporated dye with a 5 ml HiTrap desalting column (GE Healthcare). The dye/VLP mixture was loaded onto the HiTrap column pre-equilibrated in TEA buffer and 0.2 ml elution fractions were collected. VLPs were detected in the elution fractions by measuring the intensity of the E protein band on a Coomassie blue-stained SDS-PAGE gel. Labelled VLPs were eluted from the desalting column in the void (excluded) volume (1.5–2.9 ml), whereas unincorporated dye was eluted with the bulk solvent, in fractions eluting 5–6 ml post-injection. The bulk solvent elution fractions were free of protein.

### Bulk fusion assays.

A solution of DiI-labelled VLPs at 2×10^12^ VLPs ml^−1^ was mixed at a 7 : 1 (v/v) ratio with each liposome suspension at 2.5 g lipids l^−1^. Fluorescence was monitored for 200 s to ensure baseline stabilization prior to the addition of the fusion-inducing buffer [0.1 M sodium acetate (pH 5.2), or 0.1 M NaH_2_/Na_2_HPO_4_ (pH 8.5) in the negative control]. Hemifusion was monitored for an additional 800 s before addition of a detergent (decyldimethylamine-*N*-oxide; Anatrace) to solubilize the viral membrane completely, as a measure of 100 % fusion. Fluorescence was monitored with a TriStar LB941 plate reader (Berthold Technologies). For pH threshold determination, SPG buffers (succinic acid, sodium dihydrogen phosphate and glycine mixed at a 2 : 7 : 7 molar ratio), titrated to various pH values between 4 and 9, were used to induce fusion. The reported pH values were determined by measuring the pH of the VLP and liposome suspension after detergent solubilization.

### Hemifusion kinetics of single flavivirus particles to planar fluid bilayers.

We designed and optimized a single-particle TIRFM fusion assay based on the experimental set-up used in a previous study on influenza virus (Floyd *et al.*, 2008, 2009). A fluid-supported bilayer was set up on a microscope coverslip inside a flow cell. Flow cells were constructed as described by Floyd *et al.* (2009). Briefly, a glass coverslip was cleaned, coated with silane, baked and coated with dextran. Two holes were drilled into a quartz plate (ChemGlass), which was coated with double-sided adhesive tape. To form the flow channel, the tape was removed from a 15×2 mm area spanning the distance between the holes in the plate. The functionalized glass coverslip was mated onto the adhesive side of the quartz plate and tubing was inserted into the holes. To create the bilayer, 0.1 µm liposomes consisting of a 4 : 2 : 3 ratio of 1-palmitoyl-2-oleoyl-*sn*-glycero-3-[phospho-rac-(1-glycerol)] : 1-palmitoyl-2-oleoyl-*sn*-glycero-3-phosphocholine : cholesterol doped with 0.05 % GD3 ganglioside (Avanti Polar Lipids) were injected into the flow cell and allowed to adsorb onto the hydrophilic dextran cushion until they spontaneously fused with each other and eventually formed a planar membrane (Nollert *et al.*, 1995). Fluorescein was also added to the target membrane as a pH sensor (fluorescein emits weakly at pH <7). The fluidity of the supported bilayer was examined by fluorescence recovery after photobleaching of a fluorescein-labelled membrane (Fig. S3).

The TIRFM instrumentation was based on an Olympus IX-71 inverted microscope. A 561 or 488 nm diode-pumped solid-state laser was passed through a neutral density filter (OD varying between 0.04 and 2.0) and an adjustable pinhole. Two biconvex lenses arranged in a 4f system focused the beam into the back aperture of a ×60/1.45 numerical aperture oil objective (Olympus), and the entry of the beam into the back aperture was adjusted to achieve total internal reflection, creating an evanescent field of approximately 75 µm in diameter. Emitted fluorescence was passed through to an iXon back-thinned EMCCD camera (Andor) for detection. Images were captured using the Solis software package (Andor). Trajectories were collected for 40–300 s (200–300 frames, 0.2–1 s exposures).
